# Effect of sulforaphane on long-term storage of rabbit semen

**DOI:** 10.1590/1984-3143-AR2023-0001

**Published:** 2023-05-29

**Authors:** Serkan Ali Akarsu, İbrahim Halil Güngör, Aslıhan Çakır Cihangiroğlu, Tutku Can Acısu, Recep Hakkı Koca, Gaffari Türk, Mustafa Sönmez, Seyfettin Gür

**Affiliations:** 1 Department of Reproduction and Artificial Insemination, Faculty of Veterinary Medicine, Ataturk University, Erzurum, Turkey; 2 Department of Reproduction and Artificial Insemination, Faculty of Veterinary Medicine, Fırat University, Elazığ, Turkey; 3 Department of Reproduction and Artificial Insemination, Faculty of Veterinary Medicine, Bingöl University, Bingöl, Turkey

**Keywords:** apoptosis, cryopreservation, oxidative stress, rabbit, semen

## Abstract

In this study, it was aimed to determine the effect of sulforaphane (SFN) on rabbit semen cryopreservation. Semen collected from animals was divided into 5 equal volumes as Control, SFN 5 µM, SFN 10 µM, SFN 25 µM and SFN 50 µM groups. Afterwards, semen analyzes were performed. According to our results, there was no statistical difference between the groups at 4°C. However after freezing thawing, the highest total motility, progressive motility and rapid spermatozoa rate was seen in the 10 µM SFN group, while the lowest was observed in the 50 µM SFN group (P<0.05). Static sperm ratio was highest in the 50 µM group, while the lowest was observed in the 10 µM SFN group. When flow cytometry results examined the rate of acrosomal damaged and dead sperm was the lowest in the 10 µM SFN group, a statistical difference was observed between the control group (P<0.05). The highest rate of sperm with high mitochondrial membrane potential was seen in the 5 µM SFN and 10 µM SFN groups. Apoptosis and ROS rates were found to be lower in the experimental groups compared to the control groups (P<0.05). As a result, SFN supplementation at a dose of 10 µM increased the quality of sperm in the freezing and thawing processes of rabbit semen. In conclusion, 10 µM SFN improved the quality of cryopreservation of rabbit semen.

## Introduction

The decrease in the ratio of motile and viable sperm after cryopreservation of rabbit semen is a major disadvantage for the preservation of genetic resources in the creation of a sperm bank (Mocé et al., 2003; Iaffaldano et al., 2012; Iaffaldano et al., 2014). This decrease in motility and viability of semen after cryopreservation in rabbits can be affected by many parameters such as diluent, cryoprotectant, concentration, freezing protocol, thawing temperature (Mocé and Vicente, 2009). Sperm freezing extenders contain a buffer and cryoprotectant(s) to prevent cell damage caused by cryogenic damage (Parrish and Foote, 1986). Antioxidant agents can be added to extenders to protect sperm cells from cryo-damage (Mohammed et al., 2021). It has been reported that antioxidants added to the freeze-thaw medium in rabbit semen protect against cryo-damage and oxidative stress (Sarıözkan et al., 2013; Domingo et al., 2018; Abdelnour et al., 2022).

Sulforaphane (SFN) is a natural compound with antioxidant, anti-proliferative and chemotherapeutic properties (Guerrero-Beltrán et al., 2012). SFN exhibits higher bioavailability than well-known antioxidants such as quercetin (Warwick et al., 2012) and curcumin (Houghton, 2019). In the study on the effect of SFN on health, it was stated that it is a powerful activator of cellular defense systems (Zhang et al., 1992). Later studies determined the curative effects of SFN in various types of cancer (Ishiura et al., 2019; Xu et al., 2019; Georgikou et al., 2020; Wu et al., 2020). SFN is a nuclear factor erythroid 2–related factor 2 (Nrf2) activator that prevents oxidative damage (Keum, 2011). In diabetic rats, SFN causes an increase in Bax/Bcl-2 expression level and a decrease in cleavage caspase 3 and 8 levels in testis tissue. (Wang et al., 2014). The protective effect of SFN in testicular damage induced by Di-N-butylphthalate (DBP) has been reported (Qin et al., 2017). SFN was also stated to reduce the rate of apoptosis and inhibit oxidative stress (Yang et al., 2018).

In the literature review, it was seen that the studies determining the effect of the addition of SFN to rabbit semen were limited. Therefore, the aim of this study is to determine the effect of SFN addition on semen cryopreservation of New Zealand rabbits.

## Methods

### Chemicals

Sulforaphane (Cas No: 4478-93-7), Annexin V FITC assay kit and JC-1 were purchased from Cayman Chemical Company (Cayman Chemical, Michigan, Ann Arbor, USA). Lectin PNA and H2DFCDA were purchased from Thermo Fisher. All other chemicals used in the study were purchased from Sigma (Sigma, Aldrich Chemical Company, Burlington, Massachusetts, USA).

### Animals, semen collection and cryopreservation process

A total of 7 New Zealand rabbits, 6 male and 1 female, were used in the study. Rabbits were obtained from Fırat University Experimental Research Center and housed in separate cages under standard laboratory conditions (22-24 °C and 55-60% relative humidity, 12 hours/12 hours light/dark cycle). During the study, rabbits were given commercial pellet feed and fresh drinking water ad libitum. Before starting the experiments, a certificate of approval (Protocol no:2021/14) was obtained from the Fırat University Animal Experiments Local Ethics Committee. Animal care and experimental protocols were conducted in accordance with the Manual for the Care and Use of Laboratory Animals. From February to March, semen samples were taken by artificial vagina and gel portions were removed once a week from the animals. Prior to pooling, sperm motility was examined at 100X magnification with a light microscope (Celestron, Torrance, California, USA) with a heating plate. Semen samples with values below 75% motility were not used in the study. Ejaculates were diluted with 1/1 Tris-egg yolk extender (250 mmol/L Tris-hydroxymethyl-aminomethane, 88 mmol/L citric acid, and 47 mmol/L glucose, %15 egg yolk, 100 µg/ml streptomycin and 100 IU/ml penicillin) and pooled. Pooled semen was divided into 5 equal volumes as 5 µM SFN group, 10 µM SFN group, 25 µM SFN group, 50 µM SFN group and control group. It was diluted to 40x106/mL spermatozoa with 1/1 tris-egg yolk containing 5% DMSO in final volume (Rosato and Iaffaldano, 2013).

After the final dilution, the temperature of the semen was gradually cooled 35 °C to 4 °C in 90 min (Küçük et al., 2021). Semen analyzes were performed by computer assisted semen analysis (Hernández et al., 2013) method (ISAS, Proiser, Buñol, Spain). Semen samples were examined with Spermtrack slide at +4 °C (Halo et al., 2021). Then, semen samples were drawn into 0.25 mL straws with the help of an automatic pipette and left for equilibration 10 min (Küçük et al., 2021). Straws were then frozen with an automatic sperm freezing device (Mini Digitcool, IMV Technologies, L'Aigle, France). As stated by Salisbury et al., frozen semen samples were thawed at 37°C for 30 sec (Küçük et al., 2021) after 2 months (Salisbury et al., 1978). Then, 10 μL of sperm samples were taken from all groups and analyzed with CASA.

### Flow cytometric analysis

#### The rate of dead sperm

The rate of dead-viable sperm was determined by dual fluorescent staining with SYBR-14/PI described by Viudes de Castro et al. (2014). Briefly, diluted semen samples were transferred to tubes containing 2.5 µL of SYBR-14 and 2.5 µL of PI and stained. Samples were incubated at 22°C for 10 min, and analyzed using flow cytometry (Beckman Coulter, Torrance, California, USA).

#### Acrosomal status

Acrosome damage rate was evaluated by flow cytometry with FITC-PNA/PI staining method with modification of the method used by Jiménez-Rabadán et al. (2015). 30 µL of semen sample was diluted in 860 µL of PBS. It was incubated with 5 µL of PNA and 2.5 µL of PI on it for 15 min. 488 nm argon ion laser and PI and FITC-PNA was used.

#### Sperm apoptosis rate

Sperm apoptosis rate was determined by annexin V staining method described by Chaveiro et al. (2007). Briefly, the sperm sample was added to the binding buffer. 100 µL of sperm suspension was placed in an eppendorf tube containing annexin V (5 µL) and propidium iodide (PI; 5 µL). It was incubated in the dark at room temperature for 15 min and then suspended in a binding buffer (200 µL). Apoptosis rate was calculated by collecting early apoptotic sperm (A+/PI-), dead, late apoptotic and early necrotic sperm (A+/P+) with modification of the method used by Pena et al. (2003).

#### Mitochondria membrane potential assay

Mitochondrial membrane potential (MMP) was determined by the method used by Abdelnour et al. (2022). Semen samples were washed with a phosphate buffer solution (PBS) and made following the steps included in the MMP assay kit (JC-1). Sperm cells (~1 x 10^6^/ml) were incubated with JC-1 for 10 min at 37°C in the dark. The sperm samples were then examined under a flow cytometry.

#### Reactive oxygen species analysis

Reactive oxygen species were detected using the method described by Kim et al. (2011) using 2,7-dichlorodihydrofluorescein diacetate (H2DCFDA; Invitrogen, Waltham, Massachusetts, USA) and counterstained with PI to assess sperm viability. Briefly diluted sperm samples were stained with 200 µM H2DCFDA and incubated at 37°C in the dark. After 55 min the sample was mixed with PI at a final concentration of 3 µM and incubated for a further 5 min for a total incubation of 1 hr. The rate of spermatozoa with damaged membrane and high intracellular H_2_O_2_ (PI+H2DCFDA+) and intact membrane high intracellular H_2_O_2_ (PI− H2DCFDA+) were added up and expressed as percentage %.

### Statistical analyzes

The results of the data obtained from the study were expressed as Mean ± S.E.M. SPSS (Version 26, SPSS, Chicago, IL) program was used to determine the significant differences in all parameters between the groups. Data were analyzed by Oneway Anova method with post hoc Tukey's test.

## Result

### CASA sperm analysis results at 4 °C

CASA sperm analysis results of all experimental groups at 4°C are presented in Table 1. Total motility and progressive motility values were found to be numerically superior in the 10 µM SFN and 25 µM SFN groups without statistical difference. There was no difference between the groups in terms of other kinematics and velocity parameters.

**Table 1:** Sperm analysis results with CASA at 4 ^o^ C degrees after equilibration.

	**Control**	**5 µM**	**10 µM**	**25 µM**	**50 µM**
**Total Motility (%)**	63.77±3.85	63.79±3.10	66.48±7.10	68.42±5.34	64.98±10.52
**Progressive Motlility (%)**	33.86±7.25	35.87±±5.87	35.94±5.23	36.48±4.92	34.60±5.80
**Rapid (%)**	48.94±7.53	42.80±10.87	46.44±7.21	51.32±9.21	48.60±11.32
**Medium (%)**	11.06±1.80	11.72±1.66	13.04±2.33	11.24±3.33	12.08±3.38
**Slow (%)**	5.82±1.70	5.82±2.29	4.98±1.66	5.88±2.19	4.28±2.56
**Static (%)**	34.20±7.03	39.72±8.50	35.52±8.78	31.58±5.34	35.02±10.52
**VCL (µm/sec)**	88.44±12.96	82.54±17.87	89.92±16.12	92.56±19.96	95.68±23.95
**VSL (µm/sec)**	37.62±4.09	37.78±5.23	40.84±8.19	39.26±6.86	42.72±14.03
**VAP (µm/sec)**	55.20±8.91	50.76±11.51	56.78±16.38	55.58±15.60	58.44±23.18
**LIN (%)**	51.30±17.64	47.20±8.83	45.90±8.91	43.58±8.75	44.04±13.16
**STR (%)**	69.96±12.98	76.14±8.84	73.82±9.50	72.74±9.21	73.74±9.24
**WOB (%)**	62.48±5.28	61.84±7.31	62.42±10.05	59.90±8.46	59.58±13.83
**ALH (µm)**	3.28±0.23	3.10±0.37	3.30±0.28	3.34±0.27	3.48±0.37
**BCF (Hz)**	8.40±1.01	8.36±1.44	8.38±1.36	8.56±1.70	8.12±0.64

VCL: curvilinear velocity; VSL: straight linear velocity; VAP: average path velocity; LIN: linearity; STR: sperm track straightness; WOB: wobble; ALH: amplitude of lateral head displacement; BCF: beat cross-frequency. No statistical difference was found in the data without superscript.

### Sperm analysis results after the freezing-thawing process

CASA sperm analysis results after freezing and thawing are presented in Table 2. While the total motility value was highest in the 10 µM SFN group, a statistical difference was found between the control, 25 µM SFN and 50 µM SFN groups (P<0.05). The highest progressive motility values were seen in the 10 µM SFN group, while the lowest was observed in the control and 50 µM SFN groups (P<0.05). While the highest rate of rapid sperm was seen in the 10 µM SFN and 25 µM SFN groups, a statistical difference was found between the other groups (P<0.05). Static sperm ratio was lowest in the 10 µM SFN group, followed by 5 µM SFN, control, 25 µM SFN and 50 µM SFN groups, respectively. However, the rate of static sperm in the 50 µM SFN group decreased significantly, and a statistical difference was found between the other groups. There was no difference between the groups in terms of other velocity parameters.

**Table 2:** Sperm analysis results with CASA after the freezing-thawing process.

	**Control**	**5 µM**	**10 µM**	**25 µM**	**50 µM**
**Total Motility (%)**	25.22±077^b^	27.70±1.66^bc^	31.26±3.54^c^	26.21±3.10^b^	19.04±3.78^a^
**Progressive Motlility (%)**	14.35±0.77^ab^	17.90±3.26^bc^	19.09±3.66^c^	17.26±2.58^bc^	11.60±2.62^a^
**Rapid (%)**	16.32±1.15^b^	16.92±3.21^b^	21.84±2.83^c^	17.86±1.70^bc^	11.14±3.22^a^
**Medium (%)**	8.25±3.23	8.65±1.80	7.30±1.34	7.26±2.18	5.54±0.96
**Slow (%)**	3.65±0.93	3.10±0.43	3.36±1.27	2.60±1.55	2.36±0.47
**Static (%)**	71.77±5.19^b^	71.30±3.02^b^	67.74±3.13^b^	72.29±3.74^b^	80.96±3.78^a^
**VCL (µm/sec)**	71.27±6.32	72.02±8.87	80.12±10.82	81.58±5.18	7.28±8.46
**VSL (µm/sec)**	34.35±7.20	28.87±3.09	30.08±3.58	34.02±0.72	31.44±.21
**VAP (µm/sec)**	40.40±6.88	35.77±3.41	31.48±12.76	51.28±20.91	37.50±3.29
**LIN (%)**	42.09±1.17	40.85±2.63	40.82±0.39	40.96±1.43	41.96±1.05
**STR (%)**	84.50±3.76	80.70±4.71	80.34±1.73	84.08±2.80	84.30±4.28
**WOB (%)**	50.42±0.70	50.78±0.82	49.26±1.57	50.03±1.31	51.32±1.42
**ALH (µm)**	2.92±0.13	3.18±0.21	3.10±1.16	3.66±0.18	3.24±0.48
**BCF (Hz)**	8.30±0.49	7.85±0.26	8.62±0.92	8.72±0.45	8.40±0.54

VCL: curvilinear velocity; VSL: straight linear velocity; VAP: average path velocity; LIN: linearity; STR: sperm track straightness; WOB: wobble; ALH: amplitude of lateral head displacement; BCF: beat cross-frequency. No statistical difference was found in the data without superscript.

### Flow cytometry analysis results

Dead sperm rate, acrosomal damage, high mitochondrial membrane potential (HMMP), apoptosis and ROS results are shown in Table 3. While the rate of dead sperm was numerically lowest in the 10 µM SFN group, there was no statistical difference between the other groups. The rate of dead sperm with acrosome damaged was the lowest in the 10 µM SFN group, furthermore, a statistical difference was observed between the control group and the 50 µM SFN group. The highest proportion of sperm with HMMP was found in the 5 µM group, followed by the 10 µM SFN and 25 µM SFN groups. While the highest rate of apoptosis was determined in the control group, a statistical difference was found between the 10 µM SFN, 25 µM SFN and 50 µM SFN groups (P<0.05). While the highest rate of ROS was seen in the control group, there was a statistically significant difference between the other experimental groups (P<0.001).

**Table 3:** Flow cytometry analysis results in frozen thawed semen.

	**Control**	**5 µM**	**10 µM**	**25 µM**	**50 µM**
**Rate of dead spermatozoa %**	66.28±2.94	64.45±1.61	62.38±4.32	66.26±1.87	63.60±1.73
**Rate of dead sperm with acrosomal damage %**	54.63±6.78b	47.55±4.11^ab^	44.18±3.77a	51.83±3.61^ab^	52.42±4.87^b^
**Rate of viable sperm with acrosomal damage %**	2.71±1.65	2.36±1.77	2.58±2.01	2.44±0.84	1.89±0.91
**High mitochondrial membrane potential rate %**	22.91±1.85^b^	27.0±4.04c*	26.68±2.23^bc*^	25.84±1.39^bc*^	17.48±1.21^a^*
**Apoptosis %**	5.90±1.47^b^	4.50±0.79^ab^	3.59±0.56^a^	3.35±1.15^a^	3.72±0.86^a^
**ROS ratio %**	22.29±8.31^a*^	5.88±3.05^b*^	4.65±2.69^b*^	7.01±4.30^b*^	7.16±2.70^b*^

Different superscript letters in the same row display significant differences between the groups: ^a. b. c^: P < 0.05; *: P<0.001.

## Discussion

Sperm have a strong antioxidant defense system against oxidative stress; however, long-term storage processes of semen reduce this potency against ROS (Ahmad et al., 2021). By adding exogenous antioxidants to sperm extenders, the defense system against ROS can be increased and sperm quality can be improved (Petruska et al., 2014). This study was to investigate the effect of sulforaphane on the cryopreservation of rabbit semen.

Evaluation of sperm motility is an indicator of sperm quality (Shibahara et al., 2004). In addition, studies report that total sperm motility has a high correlation with fertility (Hagen et al., 2002; Lavara et al., 2005; Castellini, 2008; Sarıözkan et al., 2014). It was reported that the addition of 200 µM vitamin e analogue to rabbit semen extender increased sperm motility after freeze-thaw (Zhu et al., 2015). Similarly, trehalose increased the total motility value after freezing and thawing (Zhu et al., 2017). In the study with curcumin, the addition of rabbit semen extender increased total and progressive motility in semen (Abdelnour et al., 2020). According to the results of our study, the addition of 10 µM SFN rabbit semen to the cryo-media was found to have a protective effect against cryo-damage, increasing total motility, progressive motility, and rapid spermatozoa ratio after freezing and thawing. Moreover, 10 µM SFN supplementation reduced the rate of acrosome-damaged dead sperm, apoptosis, and ROS. The addition of 5 µM SFN and 10 µM SFN to the semen extender significantly increased the proportion of spermatozoa with HMMP.

The sperm membrane is the main organelle that is damaged during cryopreservation and short-term storage of semen. Therefore, it is necessary to protect the sperm membrane especially against ROS (Bansal and Bilaspuri, 2011). Cryopreservation of rabbit semen increases the rate of ROS in spermatozoa (Zhu et al., 2015). Excess ROS causes oxidative stress by disrupting the antioxidant balance. ROS accumulation in spermatozoa causes LPO and causes loss of membrane integrity (Zhu et al., 2017). ROS level increase reduces sperm motility by affecting membrane damage, apoptosis, mitochondrial membrane potential (Lopes et al., 1998; Sanocka and Kurpisz, 2004). ROS are determined by H2DFCDA in flow cytometry in rabbit semen (Johinke et al., 2015). In a study addition of SFN to 5 µM semen extender in human sperm cryopreservation improved sperm quality, decreased ROS level and protected plasma membrane integrity (Valipour et al., 2020). Another study reported that the added antioxidants of rabbit semen extender did not affect sperm quality (Maya-Soriano et al., 2015). In our study results suggest that ROS is inhibited by SFN at effective doses in cryopreservation, leading to an increase in sperm motility. It is thought that SFN reduces cryopreservation-induced ROS and accordingly increases sperm motility.

Apoptosis is shown among the main cause of DNA damage in sperm during spermatogenesis (Vaux and Korsmeyer, 1999). Studies have linked high levels of apoptosis with low fertility in animals (Dogan et al., 2013). In a study, it was determined that curcumin inhibited apoptosis in semen (Abdelnour et al., 2020). In our study, supplementation of 10 µM SFN, 25 µM SFN and 50 µM SFN had a lower apoptosis rate than the control group. It is stated that there is a positive relationship between apoptosis and sperm dysfunction, which increases with oxidative stress in patients with infertility problems (Wang et al., 2003).

Decrease in mitochondrial activity and sperm motility limit mitochondrial-ROS production and accordingly oxidative damage in fertilization (Johinke et al., 2014). The decreased ΔΨm potential is a sensitive indicator of mitochondrial damage by measuring cellular retention of the fluorescent probe of JC-1 (Zhu et al., 2015). In our study, the highest HMMP ratio was seen in the 5 µM SFN, 10 µM SFN and 25 µM SFN groups, respectively. Interestingly, the lowest HMMP ratio was observed in the 50 µM SFN group. This was thought to be due to SFN creating a toxic effect at high doses, slowing sperm motility and increasing acrosomal damage.

## Conclusion

Sulforaphane supplementation of the semen extender has a dose-dependent positive effect on sperm motility, velocity parameters, mitochondrial membrane potential, ROS rate, and apoptosis in rabbits after cryopreservation. Addition of 10 µM dose of sulforaphane to rabbit semen extender was beneficial for long-term storage of rabbit semen.

## Data Availability

The data used to support the findings of this study are available from the corresponding author upon request.
